# Cavitation assisted endoplasmic reticulum targeted sonodynamic droplets to enhanced anti-PD-L1 immunotherapy in pancreatic cancer

**DOI:** 10.1186/s12951-022-01459-w

**Published:** 2022-06-16

**Authors:** Jifan Chen, Liting Feng, Peile Jin, Jiaxin Shen, Jiayue Lu, Yue Song, Guowei Wang, Qin Chen, Deyi Huang, Ying Zhang, Chao Zhang, Youfeng Xu, Pintong Huang

**Affiliations:** 1 Department of Ultrasound in Medicine, The Second Affiliated Hospital of Zhejiang University School of Medicine, Zhejiang University, Hangzhou, 310000 China; 2 Department of Ultrasound, Ningbo First Hospital, Ningbo, 315000 China; 3 Research Center of Ultrasound in Medicine and Biomedical Engineering, The Second Affiliated Hospital of Zhejiang University School of Medicine, Zhejiang University, Hangzhou, 310009 China; 4 Department of Ultrasound, Sichuan Provincial People’s Hospital, University of Electronic Science and Technology of China, Chengdu, 610000 China; 5 Department of Clinical Laboratory, Second Affiliated Hospital, Zhejiang University School of Medicine, Hangzhou, 310009 China; 6 Department of Ultrasound, Yuhuan People’s Hospital, Taizhou, 317600 China; 7 Research Center for Life Science and Human Health, Binjiang Institute of Zhejiang University, Hangzhou, 310053 China

**Keywords:** Sonodynamic therapy, Endoplasmic reticulum, Immunogenic cell death, Ultrasound cavitation, Nanodroplets

## Abstract

**Background:**

Sonodynamic therapy (SDT) induces immunogenic cell death (ICD) in tumors and promises to play an assistive role in immunotherapy in pancreatic cancer. However, the short half-life and limited diffusion distance of reactive oxygen species (ROS) impair ICD induction, especially in tumors with relatively poor blood perfusion and dense stroma.

**Results:**

To address this problem, we fabricated cavitation-assisted endoplasmic reticulum (ER) targeted sonodynamic nanodroplets (PMPS NDs, 329 nm). The good sonodynamic effect and precise endoplasmic reticulum target effect was verified. After intravenous injection, the cRGD peptide modified nanodroplets initially aggregated around the tumor vascular endothelial cells. Stimulated by ultrasound, the liquid-to-gas bubbles began to oscillate and cavitate. This acoustic droplet evaporation strategy facilitated transport of the nanoparticle across the vessel, with deep penetration. This loosened the tumor stroma and facilitated accumulation and penetration of loaded sonosensitizer after 6 h. The modified sonosensitizer can selectively accumulate in the ER to generate a large amount of ROS in situ, inducing potent ER stress, amplified ICD and dendritic cell maturation in vitro and in vivo. Furthermore, the elevated antitumor effect of SDT plus anti-PD-L1 immunotherapy was verified using an orthotopic tumor model.

**Conclusions:**

This study reports a cavitation assisted ER targeted sonodynamic therapy that can enhance the effect of anti-PD-L1 immunotherapy effectively in orthotopic and distant pancreatic cancer.

**Graphical Abstract:**

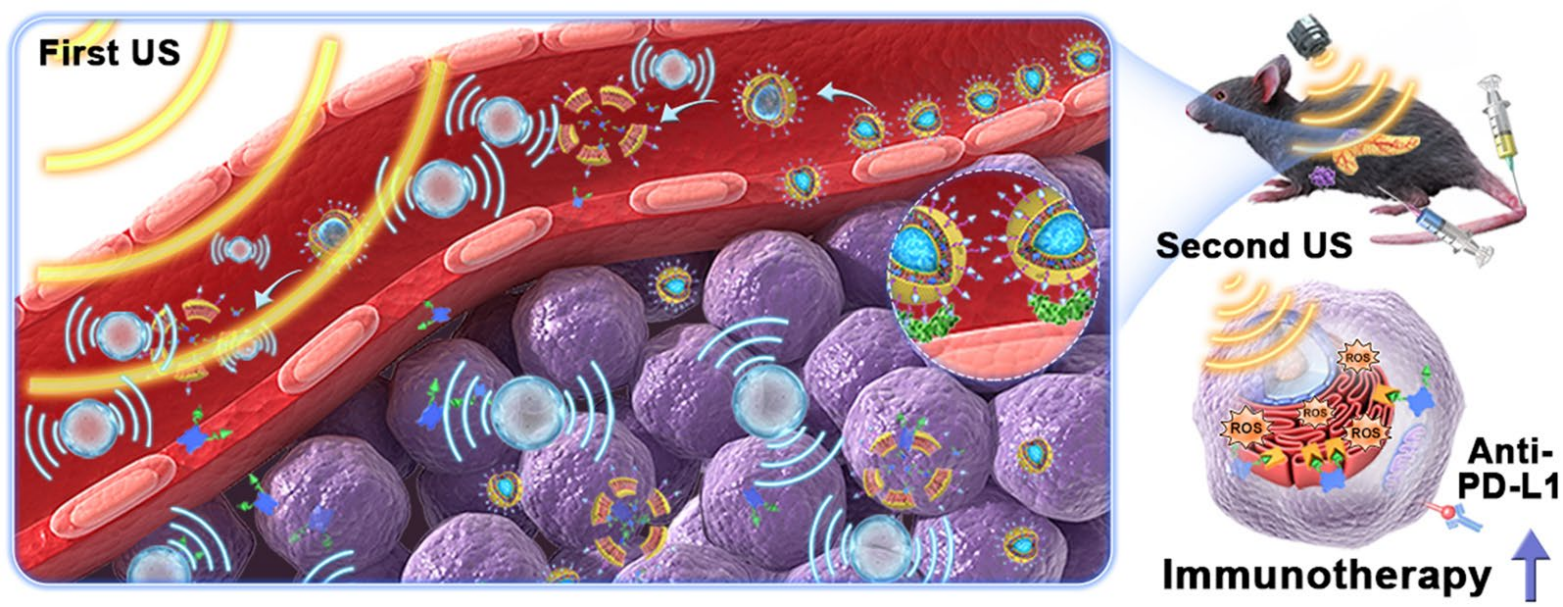

**Supplementary Information:**

The online version contains supplementary material available at 10.1186/s12951-022-01459-w.

## Background

Immune checkpoint inhibitors, targeting cytotoxic T-lymphocyte-associated protein 4 (CTLA-4) and the programmed cell death protein-1 (PD-1)/programmed cell death ligand-1 (PD-L1) pathways have shown remarkable potential in several types of cancer [1]. However, checkpoint inhibition monotherapy has failed to elicit efficacy in patients with pancreatic cancer [2]. In pancreatic cancer, lack of antigenicity and abundant immunosuppressive mechanisms, such as a low degree of mutation, high PD-L1 expression, and low MHC-1 expression, were reported [3, 4]. The tumor high intensity stroma and fibrosis are a bottleneck for ideal drug delivery and penetration [5]. Some strategies for increasing the efficacy of immunotherapy and for ideal drug delivery have been investigated by researchers in pancreatic cancer [6–9].

Sonodynamic therapy (SDT) can induce a different reaction in the tumor cell, for example, apoptosis [10], necrosis [11], and autophagy [12]. Recently, researchers have focused the immunity-related death such as necroptosis [13] and immunogenic cell death (ICD) [14–16]. ICD comprises the release of damage-associated molecular patterns (DAMPs) from dying tumor cells that activate tumor-specific immune responses [17]. Thus, some researchers combine SDT with immunotherapy to eliminate primary tumors, inhibit metastases, and elicit long-term anticancer efficacy with a remote, noninvasive and safety feature [8, 18, 19].

However, the reactive oxygen species (ROS) such as singlet oxygen (^1^O_2_) generated in SDT may have limited efficacy [20]. Anti-ROS components such as antioxidant enzymes and small-molecule antioxidant compounds in vivo could counterbalance the increased ROS [21], while the effective distance and duration time of ROS are relatively short within the cellular milieu [22]. Furthermore, the adequate drug delivery in pancreatic cancer is hindered by relatively poor blood perfusion and dense stroma.

**Scheme 1:**
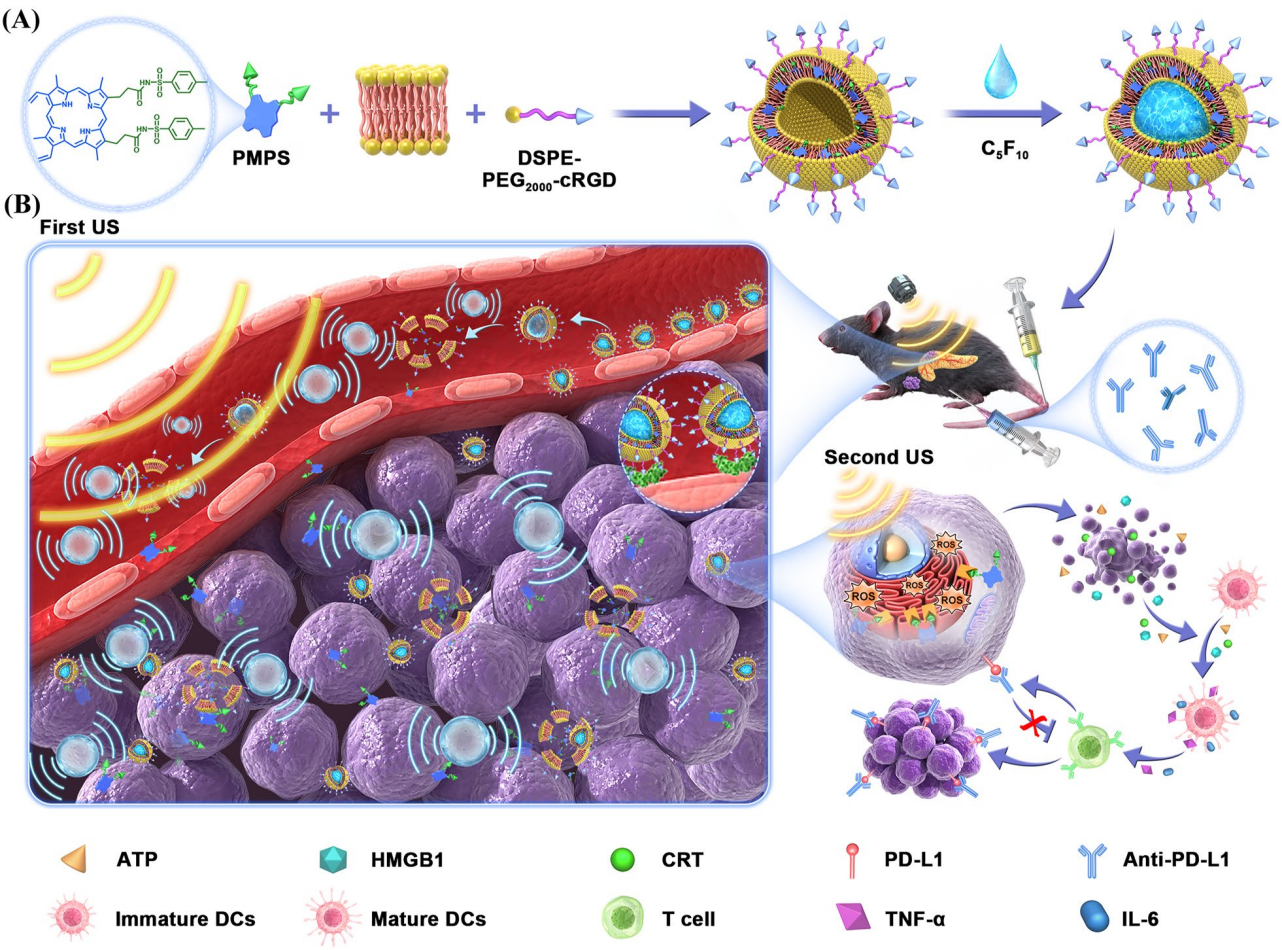
**A** Schematic illustration of the synthesis route of PMPS NDs. **B** The in vivo cavitation-assisted ER targeted sonodynamic therapy and aPD-L1 immunotherapy at orthotopic and distant tumor models

To tackle these problems, a cavitation assisted endoplasmic reticulum (ER) targeted sonodynamic therapy can facilitate drug accumulation and penetration, thus elevating ER stress [23, 24] in situ and boosting the efficacy of immunotherapy. Firstly, the tumor vascular targeted acoustic droplets evaporation (ADV) increases the aggregation and penetration of nanodroplets in the tumor microenvironment [25]. Under ultrasound (US) irradiation, the perfluoropentane transfers from the liquid phase to the gas phase and cavitates. Meanwhile, more nanoparticles are transported across the open vascular wall during the non-radiation period, and then pushed deep inside the tumor during the irradiation period. The dense tumor stroma is loosened after US, facilitating sonosensitizer diffusion and internalization by the tumor cell. Then, the modified sonosensitizer can selectively accumulate in the ER to generate a large amount of ROS in situ, inducing potent ER stress and amplified ICD. Herein, this study fabricates a cavitation-assisted ER-targeted sonodynamic droplets (PMPS NDs), achieving deep penetration and in situ sonodynamic therapy to enhance the effect of Anti-PD-L1 (aPD-L1) immunotherapy in orthotopic and distant pancreatic cancer (Scheme 1).

## Result and discussion

### Characterization of PMPS NDs

**Fig. 1:**
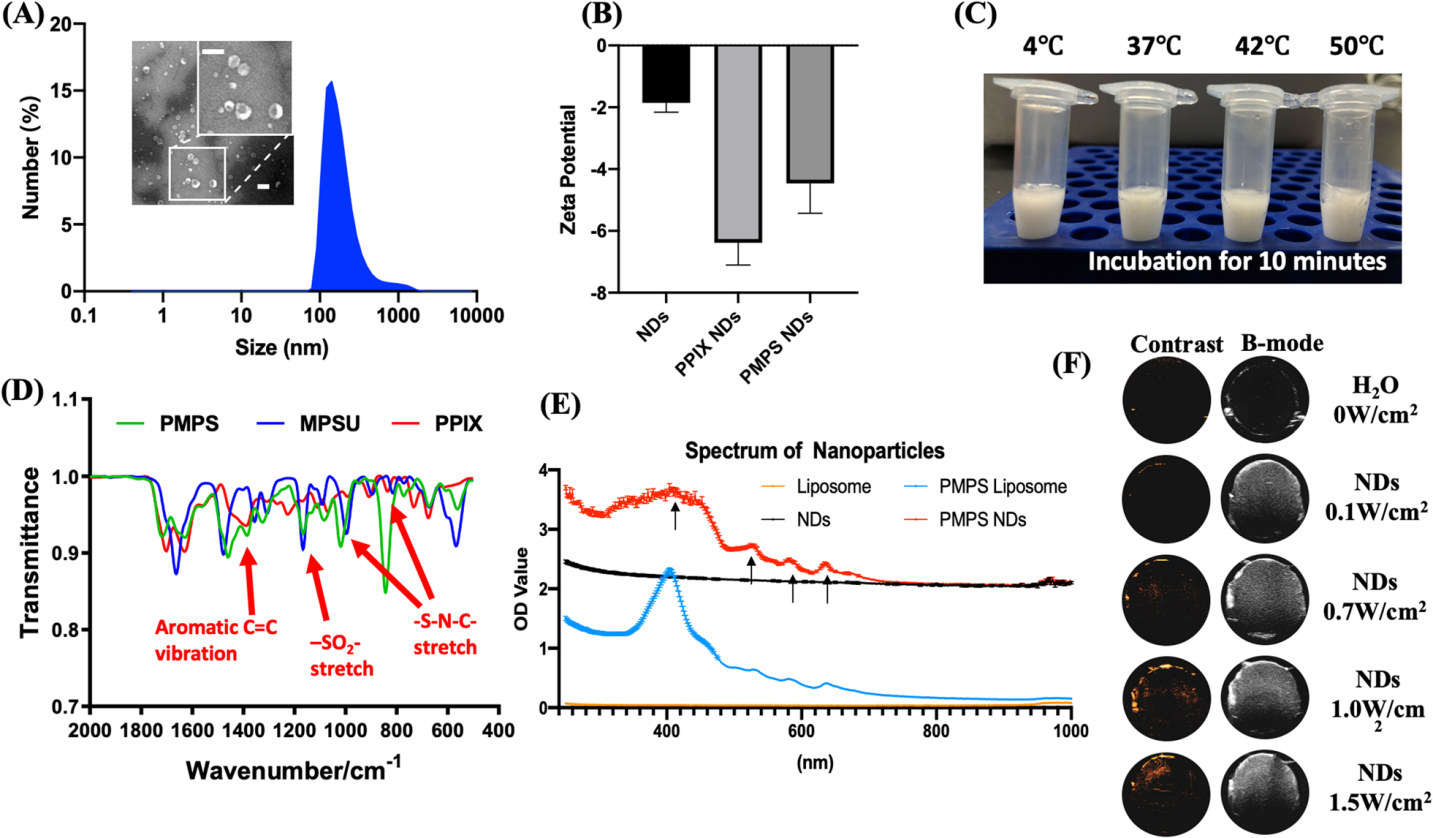
Characterization of PMPS NDs. **A** The size of nanodroplet by DLS and TEM (Scale bar: 500 nm). **B**The zeta potential of three NDs. **C** Digital picture of NDs in different temperature, **D** the infrared spectrum (**D**) and US-vis spectrum (**E**) of PMPS. **F** the contrast and B mode image by different acoustic parameters

PMPS was synthesized by amidation of protoporphyrin IX (PPIX) with 4-methylphenylsulfonylurea (MPSU), in the presence of BOP reagent and triethylamine (TEA). The MPSU binds to the sulfonylurea receptors on ER membrane to achieve the endoplasmic reticulum targeted capability [26]. The synthesized PMPS NDs had a hydrodynamic diameter of 329.7 ± 62 nm by dynamic light scattering (DLS), and a round morphology by transmission electron microscopy (TEM) (Fig. 1A). The zeta potentials of nanodroplets (NDs), PPIX NDs and PMPS NDs were − 1.85 ± 0.31, − 6.38 ± 0.72 and − 4.46 ± 0.97 mV, respectively (Fig. 1B). After incubation at different temperatures (4, 37, 42, 50 °C) for 10 min, the NDs generated less bubbles in 4 and 37 °C, than at 42 and 50 °C (Fig. 1C). In addition, we verified the conjugated PPIX and MPSU by infrared spectrum (Fig. 1D) and nuclear magnetic resonance (NMR) (Additional file 1: Figure S1). The invert peak of –SO_2_– and –S–N–C– stretch were evident in the PMPS infrared spectrum. In addition, we confirmed the successful loading of PMPS in NDs by ultraviolet and visible (UV–vis) spectroscopy (Fig. 1E), with a load ratio of 7.4% (equal load rate of PPIX 4.2%) and encapsulation efficiency of 81.3%. The successful loading of perfluoropentane (C_5_F_10_) was verified by increasing the OD value in the UV spectrum.

### Assessment of the acoustic droplet vaporization (ADV)

We compared the ADV effect in NDs loaded with perfluoropentane and perfluorooctane using an ultrasound image system (Additional file 1: Figure S2A). After irradiation with 0.1 W/cm^2^, 0.7 W/cm^2^, 1.0 W/cm^2^ and 1.5 W/cm^2^ ultrasound, the signal intensity in contrast ultrasound mode showed an obvious increase in PMPS NDs (Fig. 1F). We observed a linear increase (Additional file 1: Figure S2B), demonstrating that the PMPS NDs have ADV effect and the intensity of ADV increases with increasing acoustic intensity.

### Assessment of the endoplasmic reticulum targeted and sonodynamic effect of PMPS NDs

**Fig. 2:**
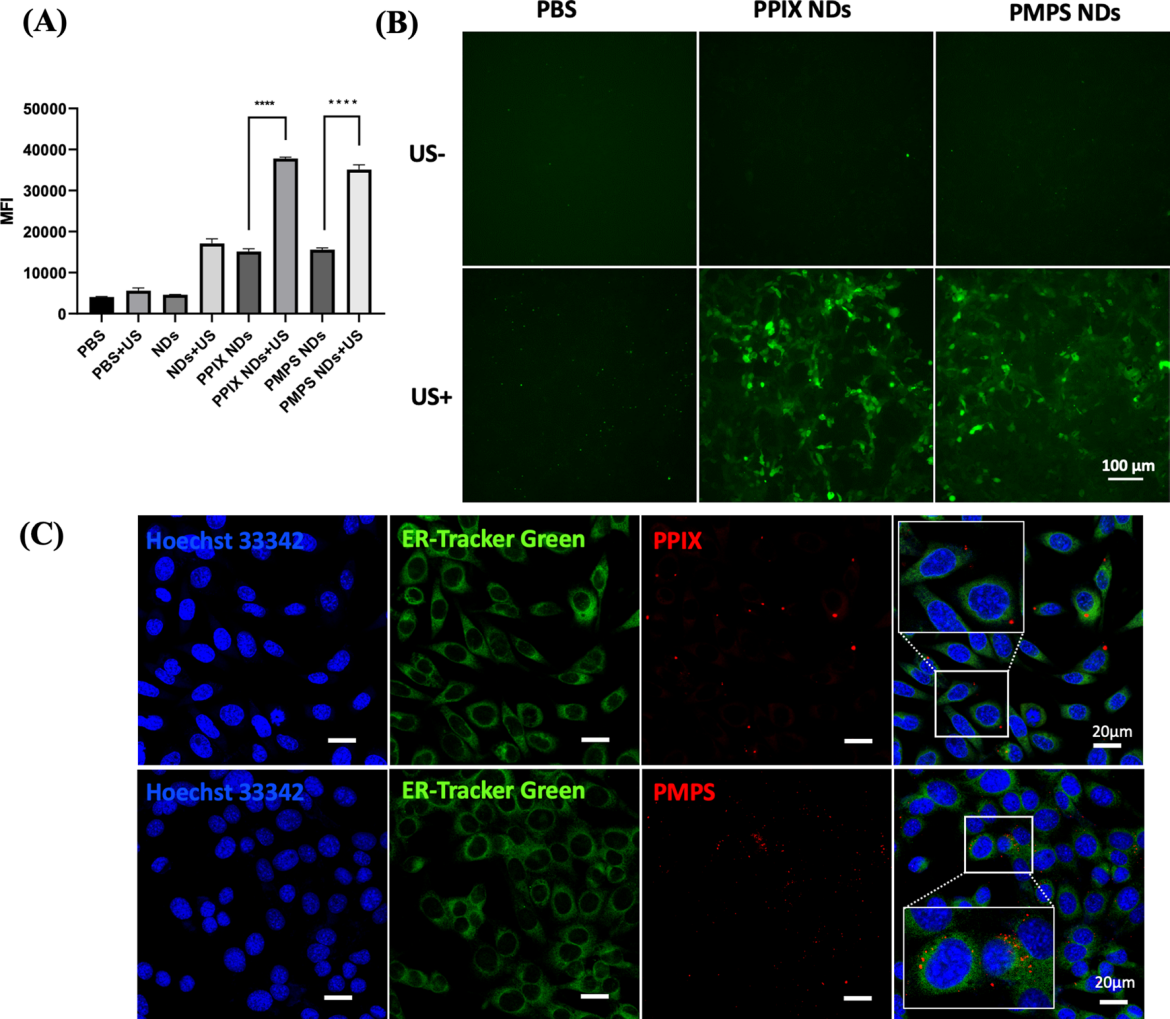
The sonodynamic and endoplasmic reticulum targeting effect in vitro. The sonodynamic effect of synthesized NDs (**A**) and cellular ROS generation (**B**) under US irradiation. **C** The subcellular location of PPIX NDs and PMPS NDs

To investigate the sonodynamic effect, we applied US (1.0 W/cm^2^) in liquid containing PBS, NDs, PPIX NDs, and PMPS NDs. No statistically significant difference was found between PBS and PBS + US groups (Fig. 2A). The cavitation effect can interpret the mildly elevated fluorescence in NDs + US. In PPIX NDs + US and PMPS NDs + US group, a vast elevated fluorescent intensity was obtained, indicating that the sonodynamic effect was achieved. Similar results were found in vitro. The tumor cells in PPIX NDs + US and PPIX + NDs + US group turned green in the fluorescent image. (Fig. 2B).

In addition, we evaluated the endoplasmic reticulum targeted effect of PMPS NDs. After incubating with cells for 6 h, we used a confocal laser fluorescent image to show the relationship between PMPS and the endoplasmic reticulum. As shown in Fig. 2C, the fluorescence of porphyrin occurred close to the endoplasmic reticulum in the PMPS NDs group. However, the distance of porphyrin fluorescence is farther in the untargeted group (PPIX NDs). This result demonstrates that PMPS could selectively accumulate into the endoplasmic reticulum with the help of conjugated MPSU and the ER-target sonodynamic therapy is achieved.

### Assessment of the antitumor effect and BMDCs maturing in vitro

**Fig. 3:**
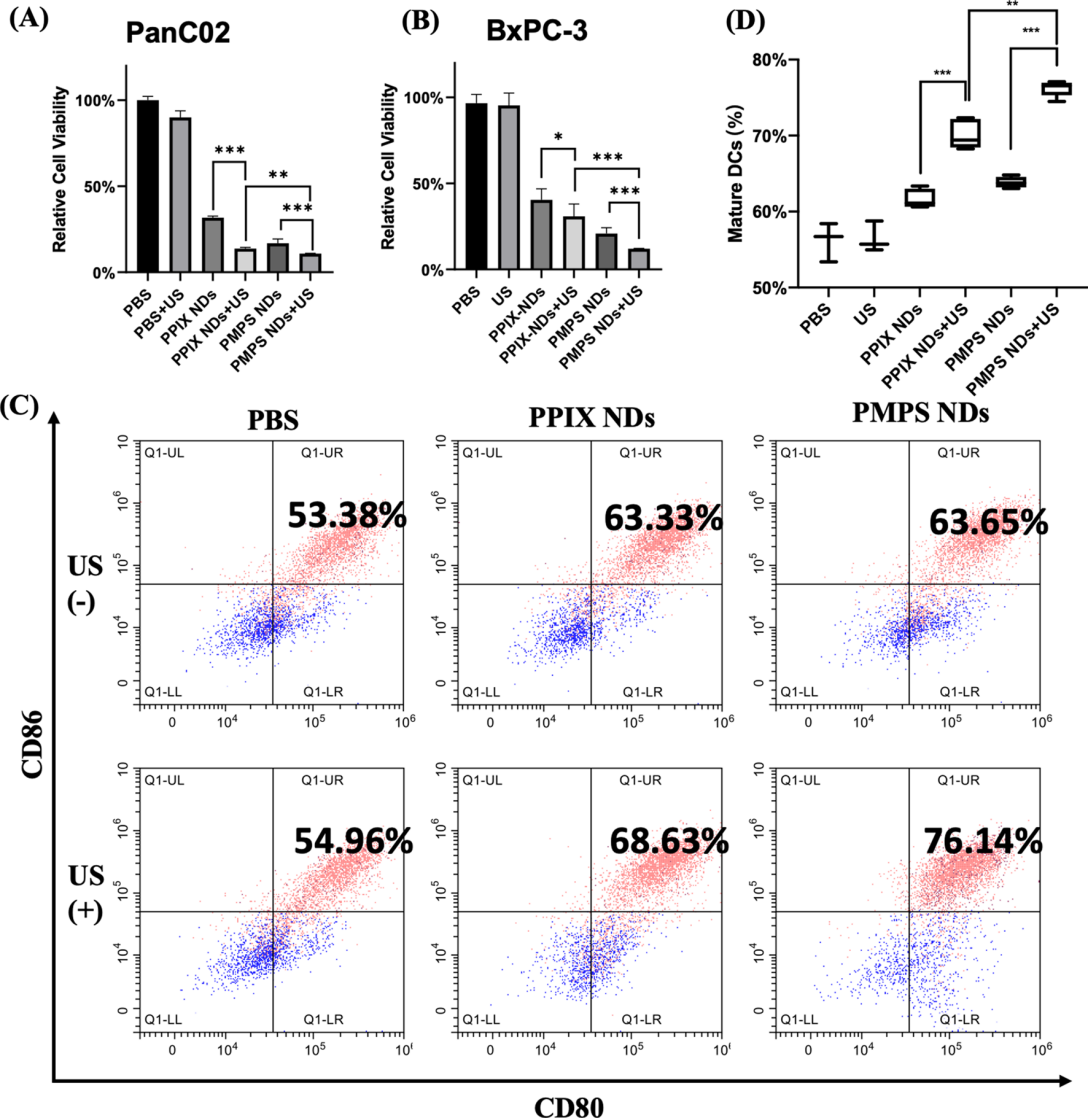
The antitumor and DC maturation effect in Vitro. **A** The anti-tumor effect evaluated using Panc02 (**B**) and BxPC-3 cell lines. **C** The DCs stained with CD80 and CD86 under different treatment and the quantitative data (**D**)

The antitumor effect of PMPS NDs + US group is highest among the other groups (PBS, PBS + US, PPIX NDs, PPIX NDs + US, PMPS NDs groups) both in PanC02 and BxPC-3 cell line (Fig. 3B) using CCK-8 kit. Besides, similar results were found in the apoptosis ratio using flow cytometry (Additional file 1: Figure S3). The PMPS ND + US group had the highest apoptosis rate (68.2%), while the control group was 3.91%. This result shows that the ER targeted sonodynamic therapy could increase the therapeutic efficacy.

To investigate the BMDCs maturation among different treatment groups, we co-cultured pre-treated PanC02 cells with bone marrow dendritic cells (BMDCs) separated from C57Bl/6 mice. After co-stimulation for 48 h, the proportion of CD86 and CD80 dual-positive BMDCs increased in PMPS NDs + US (76.22% ± 1.03%) and PPIX NDs + US group (70.14% ± 1.92%), compared with no US groups (63.84% ± 0.73%, 61.68% ± 1.25% respectively) (Fig. 3C, D). These results demonstrate that tumor cells treated with sonodynamic therapy can induce ICD and promote BMDC maturation. Moreover, the endoplasmic reticulum targeted strategy could have a higher antitumor effect, potently stimulating BMDC maturation, compared with the non-targeted group.

### Assessment of CRT, HMGB-1 Expression in vitro

**Fig. 4:**
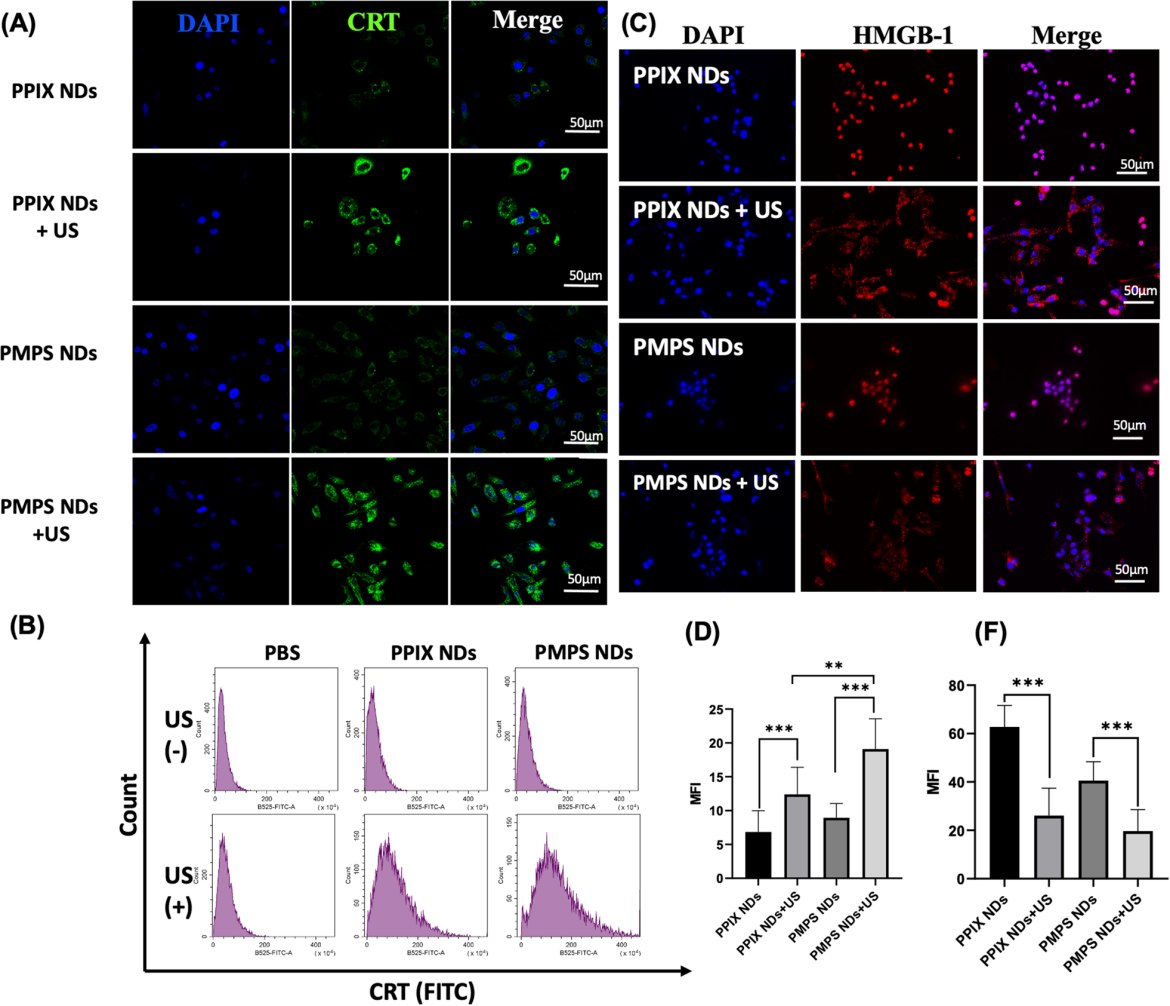
The CRT and HMGB-1 expression in immunogenic cell death. The IF (**A**) and FCM (**B**) result of CRT expression. The IF of HMGB-1 (**C**). The quantitative data of CRT (**E**) and HMGB-1 (**F**)

The expression of ICD indicators such as calreticulin (CRT) and high mobility group box 1 (HMGB-1) were investigated in vitro. The expression of CRT was measured using a fluorescent microscope (Fig. 4A) and flow cytometry (FCM) (Fig. 4B). We found higher CRT expression in the sonodynamic group (PPIX NDs + US and PMPS NDs + US), and the location of CRT shifted compared with the non-US groups (PPIX NDs and PMPS NDs) (Fig. 4A). The expression of CRT in the PMPS NDs + US group was higher than the non-ER target group (PPIX NDs + US) (Fig. 4d). In the sonodynamic therapy groups (PPIX NDs + US and PMPS NDs + US), the HMGB-1 translocated from the nucleus into the cytoplasm, and the fluorescent intensity was lower in PMPS NDs + US than PPIX NDs + US group (Fig. 4C, F). This phenomenon represents the process of HMGB-1 translocating from nuclear and then extracellular released. The above results demonstrate that the ER-targeted sonodynamic strategy can induce potent ER stress, promote DAMPs release, and thus, elevate the ICD.

### Assessment of the enhanced accumulation by ADV effect in vivo

**Fig. 5:**
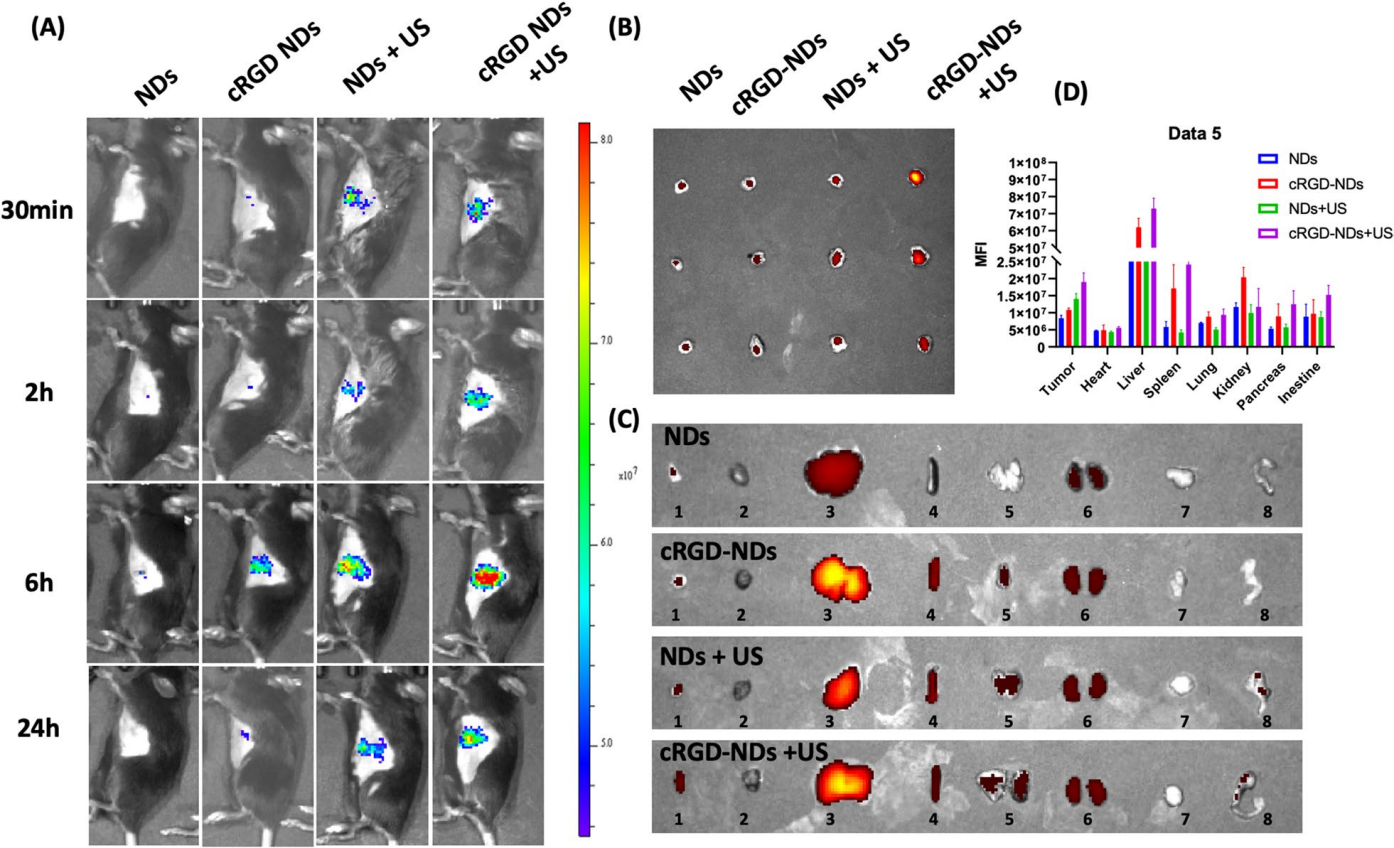
The Fluorescence images of cRGD and acoustic droplet evaporation. **A** In vivo fluorescence images to investigate the synergistic drug accumulation and penetration by tumor vascular targeted and ADV strategy. **B** ex vivo distribution of tumor (**B**) and main organ (**C**). The quantitative data was displayed in (**D**). 1: Tumor 2: heart, 3: Liver, 4: Spleen, 5: Lung, 6: Kidney, 7: Pancreas, 8: Intestine

For in vivo study, we use cRGD, a well-known tumor vascular target peptide, to generated ADV effect closely to tumor vessels. As shown in Fig. 5A, B, comparing with cRGD NDs and NDs + US (non-targeted ADV effect) group, cRGD NDs + US group accumulated the most prominent cy5.5 fluorescent signal in the tumor site. cRGD conjugated NDs aggregated near the tumor vascular endothelial cells (TVEC), and then the contained C_5_F_10_ liquid evaporated under US irradiation. The ultrasound cavitation can facilitate NDs through the vessel wall. The radiative force generated from US can also push the NDs far from tumor vasculature and accumulate deep inside the tumor. This strategy could elevate the penetration and accumulation of the loaded drug in the tumor by a synergy effect. However, in Fig. 5C, D, except for the tumor, the cRGD NDs increased the drug accumulation in hyper-vascular organs. In this case, no US was applied in normal tissues, and the safety of PMPS NDs was verified by morphology (Hematoxylin & Eosin) and blood biochemistry Additional file 1: Figure S4.

### Assessment of the DCs maturation and Cytokine release in Orthotopic Pancreatic Carcinoma Model

**Fig. 6:**
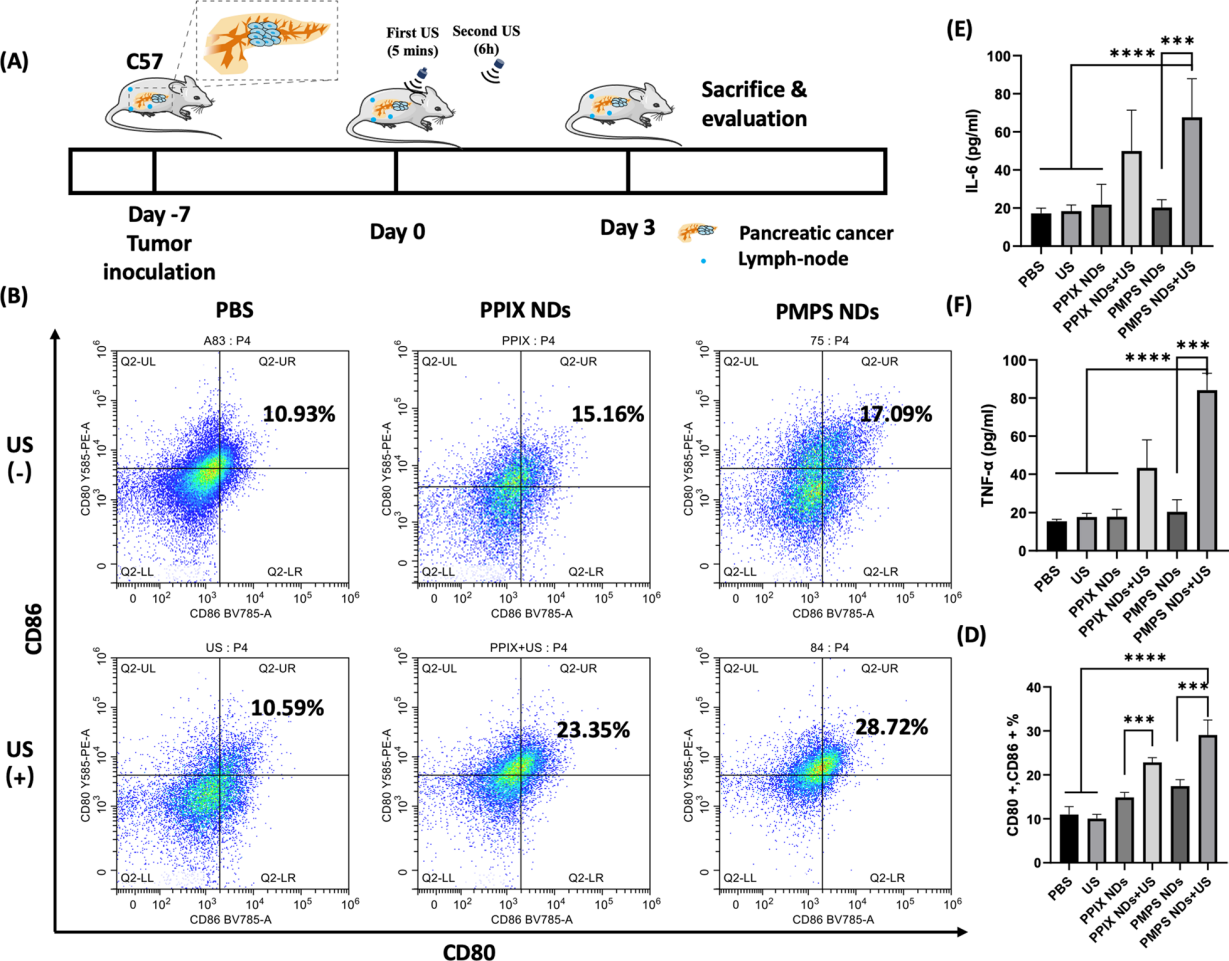
In vivo SDT for promoting DC maturation and stimulating the expression of proinflammatory cytokines. **A** Schematic illustration of SDT therapy to enhance DC maturation in orthotopic tumor models; We administrate NDs at Day0, first US at 5 min post injection for enhanced drug accumulation and second US at 6 h post-injection for sonodynamic effect. **B** DC maturation in the tumor-draining lymph nodes induced by SDT on mice bearing PanC02 tumors, as assessed by flow cytometry after staining with CD11c, CD80, CD86 and live dead and the quantitative data was displayed in (**D**); Pro-inflammatory cytokine levels of IL-6 (**E**) and TNF-α (**F**) in serum from mice were measured at Day 3 after SDT treatment

We further verify DCs maturation and cytokine release in vivo using an orthotopic pancreatic carcinoma model. The therapeutic protocol was displayed in Fig. 6A. After intravenous injection, we applied the first pulsed US irradiation to elevate the tumor's drug accumulation and penetration. After 6 h, the secondary US irradiation was applied to realize the sonodynamic therapy. The tumor-draining lymph nodes, such as pancreatic, mesenteric, inguinal, and lumbar lymph nodes, were carefully resected at Day 3. The live/death, CD11c, CD80, and CD86 were stained. The number of CD80 and CD86 dual-positive DCs in the PMPS NDs + US group (29.09% ± 3.39%) was higher than the other groups. Furthermore, we collect the blood of mice to test the IL—6 and TNF—α levels in serum. The ER-target group has higher IL-6 and TNF-α levels, compared with the non-target group (Fig. 6E, F). These results demonstrate that the treatment of PMPS NDs + US can stimulate DC maturation effectively and show promise in combination with aPD-L1 therapy.

### Assessment of the synergistic effect of PMPS NDs and aPD-L1 immunotherapy

**Fig. 7:**
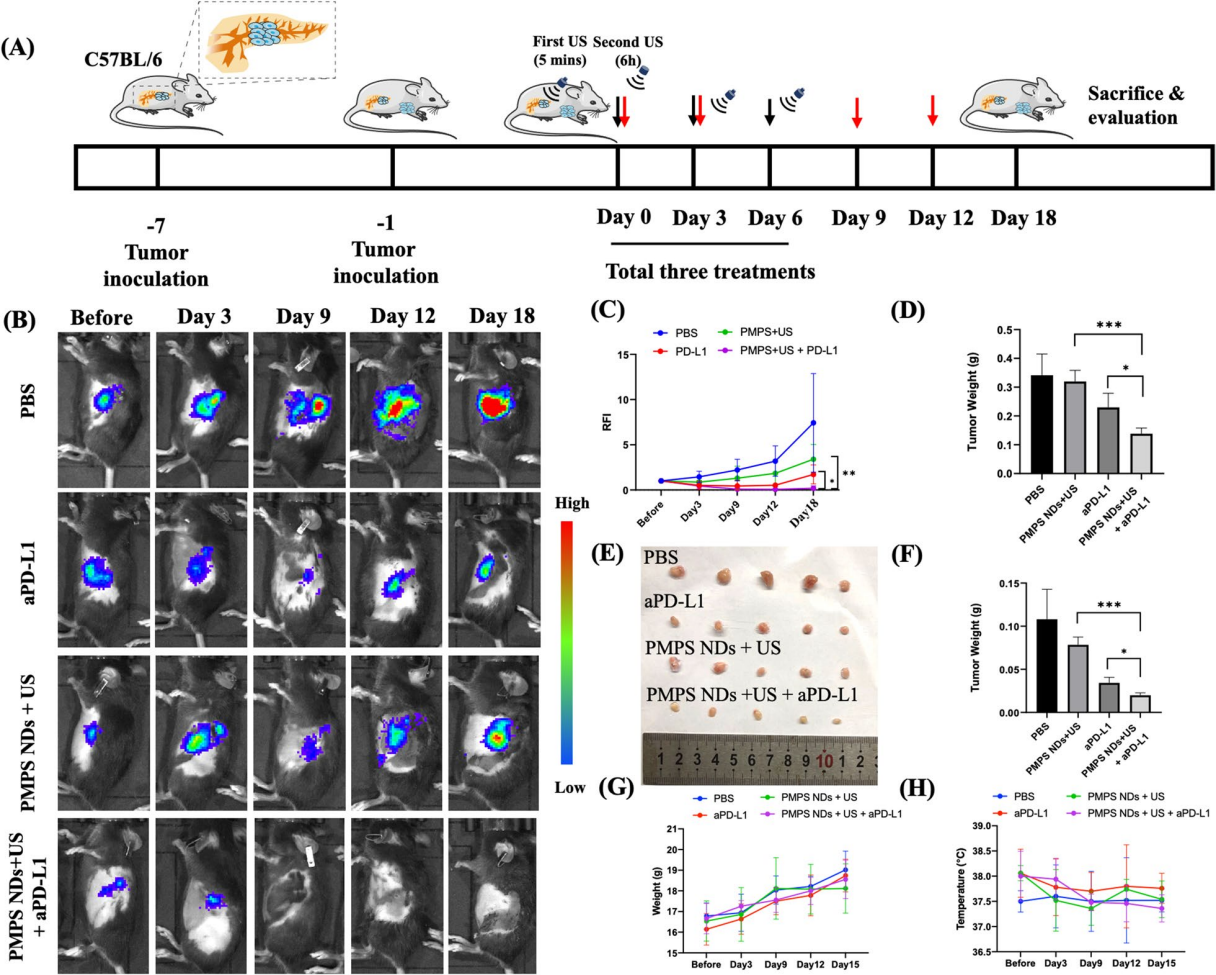
Antitumor effect of SDT plus aPD-L1 immunotherapy in orthotopic tumor models. **A** Schematic illustration of SDT and aPD-L1 combination therapy to inhibit tumor growth at orthotopic and distant tumor models; **B** In vivo fluorescence images to show the therapeutic effect on orthotopic tumor models and the quantitative data of fluorescence intensity (**C**) and tumor weight (**D**); The digital photo (**E**) and the weight of distant tumor (**F**); mice weight (**G**) and temperature (**H**) during the follow-up times

To investigate whether the Cavitation Assisted ER-targeted Sonodynamic Droplets (PMPS NDs) could elevate the therapeutic effect of anti-PD-L1 (aPD-L1) and show the ideal immunity response in vivo, we built an orthotopic and distant subcutaneous tumor model using the PanC02-Luci cell line (Fig. 7A). After seven days of inoculation of the orthotopic tumor, we administered PBS, PMPS NDs by tail vein, and the aPD-L1 antibody by intraperitoneal injection. As per a previous protocol, we applied the first US at 5 min post-injection to enhance the drug accumulation and penetration, and the second US at 6 h post-injection for sonodynamic therapy. On Day 3, the tumor photons count in the aPD-L1, and PMPS + US + aPD-L1 group decreased. During the three times of treatment (Day 0–Day 9), the tumor photons count decreased gradually in the aPD-L1 and PMPS + US + aPD-L1 groups. On Day 12, the tumor photons count in PMPS NDs + US + aPD-L1 group remained low. However, the tumor photons increased in the aPD-L1 group (Fig. 7B, C). On Day 18, the orthotopic tumor was resected for sections and dissolved for immunity FCM analysis. The distant subcutaneous tumor was resected to take the digital photo (Fig. 7E). The PMPS NDs + US + aPD-L1 group's tumor weight was lowest compared with the other groups both in the orthotopic and distant tumor (Fig. 7D, E). No obvious tumor weight loss or temperature changes were found in the whole study (Fig. 7G, H).

These results demonstrate that the synergistic strategy combining ER targeted sonodynamic therapy with aPD-L1 immunotherapy was achieved, not only in the orthotopic tumor, but also in the distant tumor. Although we only irradiated the orthotopic tumor, the SDT could help the antitumor immunotherapy in the whole body.

### Assessment of the immune-response and antitumor effect ex vivo

**Fig. 8:**
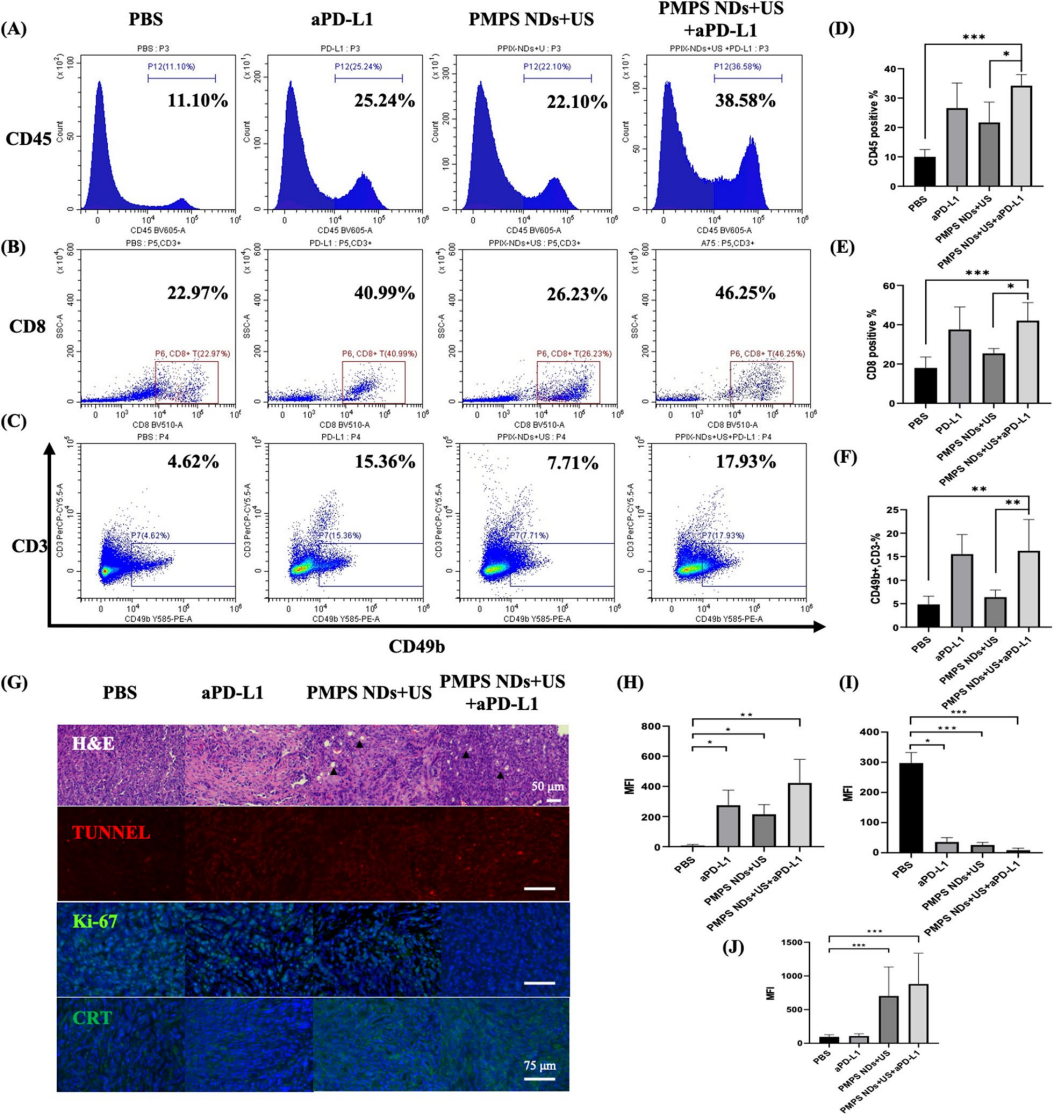
SDT plus aPD-L1 therapy activating systematic antitumor immunity. **A** Representative flow-cytometry plots showing the tumor-infiltrating leucocyte cells, including CD45 + cells (CD45 +) (**A**), CD8 + T cells (CD45 + CD3 + CD8 +) (**B**) and NK cells (CD45 + CD3-CD49b +) (**C**) in orthotopic tumors and the quantitative data (**D**–**F**). The tumor sections were stained using H&E, TUNNEL, Ki-67, and CRT (**G**) and the quantitative data (**H**–**J**)

Finally, we evaluated the immunity response in orthotopic tumor. After treatment in PBS, aPD-L1, PMPS NDs + US, PMPS NDs + US + aPD-L1 groups, the proportion of CD45 positive cell was 10.02% ± 2.45%, 26.59% ± 8.51%, 21.71% ± 6.89% and 34.21% ± 3.79%, respectively (Fig. 8A, D). The proportion of CD8 positive cell in CD3 positive & CD45 positive cell in aPD-L1 group and PMPS NDs + US group were 37.58% ± 11.43% and 40.73% ± 10.80%, respectively, compared with the PBS (18.01% ± 5.54%) and PMPS NDs + US (26.54% ± 4.62%) groups (Fig. 8B, E). Furthermore, the elevated NK cell in tumor, the proportion of CD3 negative and CD49b positive cell in aPD-L1 and PMPS NDs + US + aPD-L1 groups were 16.32% ± 4.49% and 17.62% ± 8.96 in CD45 positive cells, which is higher than PBS (4.84% ± 1.78%) and PMPS + ND + US (17.62% ± 8.96%) groups (Fig. 8C, F).

Hematoxylin and eosin (H&E) staining of the tumor tissues of the synergistically treated mice (PMPS NDs + US + aPD-L1 group) showed evidence of tumor cell death and some vacuolation in tissues that might cause by the ADV effect (Fig. 8G). The more TUNNEL positive cells, the lower Ki-67 expression was found in the combined therapy group (PMPS NDs + US + aPD-L1group). These results demonstrate the antitumor efficacy of combined PPIX NDs + US and immunotherapy. In addition, we also investigated the CRT expression in the tumor's section. A higher expression of CRT was found, supporting the result of ICD in the sonodynamic therapy group.

These results demonstrate that the efficacy of synergistic strategy can be explained by the elevated immune response in the tumor site, and especially the immunity response in CD8 positive T cells and NK cells.

## Conclusion

In summary, we have successfully synthesized the cavitation-assisted endoplasmic reticulum targeted sonodynamic droplets (PMPS NDs) for the first time. The US sequence was designed to achieve cavitation-assisted drug delivery and in situ sonodynamic therapy. After intravenous administration, the NDs aggregate around the tumor microenvironment and are evaporated by US irradiation, aiding transport of the NDs across the vessel wall during the non-irradiation interval. The bubbles generated by the US irradiation serve to loosen the tumor stroma, and the NDs can be pushed deep inside, increasing the drug accumulation and penetration. The released drug can target the tumor ER for decreasing the ROS's effective distance. Then, the second US irradiation is applied to achieve in situ sonodynamic therapy to induce the ER stress and ICD. Furthermore, the aPD-L1 antibody was injected combined with PMPS NDs + US therapy. The synergistic effect of combined therapy was verified in this study.

## Materials and methods

### Materials and reagents

The protoporphyrin IX, 4-methylphenylsulfonylurea and BOP reagent were purchased from Macklin (Shanghai, China), Yuanye Biotech (Shanghai, China) and Tensus Biotech (Shanghai, China). 1,2-Dioleoyl-sn-glycero-3-phosphoethanolamine (DOPE) were purchased from Avanti Polar Lipids Company (Alabaster, AL, USA). Cholesteryl hemisuccinate (CHEMS) was purchased from Tokyo Chemical Industry Co., Ltd (Shanghai, China). 1,2-Distearoyl-sn-glycero-3-phosphoethanol- amine N-[methoxy(polyethylene glycol)-2000] (DSPE-PEG) was purchased from Laysan Bio. Inc (USA). DSPE-PEG2000-cRGD was purchased from Xi’an ruixi Biotech Co., Ltd.

### Cell lines and animals

Panc02 cells were obtained from the Shanghai institute of biological sciences, the Chinese academy of sciences. Panc02-Luci cells were obtained as a gift from Youqin Shen’s lab in Zhejiang University. BALB/c Nude mice were bought from Shanghai SLAC Laboratory Animal Co., Ltd.

### Preparation of PMPS

PMPS was synthesized by amidation of protoporphyrin IX with 4-methylphenylsulfonylurea (MPSU) with BOP reagent and triethylamine (TEA). Typically, MPSU (85.7 mg, 0.4 mmol), BOP (176.9 mg, 0.8 mmol) and TEA (55.6 μL, 0.4 mmol) were dissolved in 5 mL dichloromethane (DCM), and the solution was dropwise added to protoporphyrin IX (56.3 mg, 0.1 mmol) in 5 mL DCM with stirring for 4 h at RT. The DCM solution was rotary evaporated, and the PMPS product was washed by 0.1 M NaOH solution (protoporphyrin IX removal) and then acetone (BOP, TEA, and MPSU removal) three times.

### Preparation of PMPS nano-droplets

PMPS (0.6 mg), DOPE (3.16 mg), DSPE-PEG2000 (2.1 mg), CHEMS (0.54 mg) were dissolved in CHCl_3_ (5 mL) and the solution were evaporated to form a thin-film in a 50 mL round-bottom flask. After hydrating by 5 mL PBS buffer, the 200 µL perfluoropropane was added in liquid on ice. Then, the above liquid was dispersed using the 50 W ultrasonic dispersion module (pulse duration, 3 s, pulse interval, 3 s) for 10 min. Finally, the PMPS nano-droplets (NDs) were obtained after centrifugation (4629 g, 5 min). The PPIX NDs and NDs were also prepared.

### Characterization of PMPS NDs

The PMPS were verified by infrared spectroscopy and magnetic resonance analysis. The morphology and size of PMPS NDs were characterized with an optical microscope, DLS and TEM. The digital photo and microscope photos of nanodroplets were recorded. The load ratio was verified by ultraviolet-vis spectrum.

### In vitro ADV capability, US imaging performance, and ROS generation of NDs

The PMPS NDs was incubated at different temperatures (4, 37, 45, 50 °C) and irradiated by ultrasound at different acoustic intensities (0, 0.1, 0.7, 1.0, 1.5 W/cm^2^). The ultrasound parameters were set as follows: (Frequency: 3 MHz; Acoustic intensity: 1.0 W/cm^2^; Duty cycle: 20%, 2 min). The intensity of contrast-enhanced ultrasound was measured by ImageJ software. The ROS generation from PBS, NDs, PMPS NDs, and PPIX NDs with/without US irradiation were measured using DCFH-DA, and the intensity of Ex488nm/Em520nm was detected using a multiple function enzyme-labeling instrument (SPARK CYTO).

### In vitro cellular distribution of PMPS NDs

The PanC02 cells were incubated with PMPS and PPIX NDs for 6 h. Then, the cellular nuclear and endoplasmic reticulum (ER) were stained by NucBlue™ and ER Tracker™ Green. (Invitrogen, Thermo Fisher Scientific) Finally, the photos of the strained cell were recorded by an invert confocal fluorescent microscope.

### Cell viability assay and ROS generation

The PanC02 and BxPC-3 cell were incubated with PBS, PMPS NDs (PPIX: 0.1 mg/ml), PPIX NDs (equal dose of PPIX: 0.1 mg/ml) for 4 h, and the culture medium were replaced. The cell viability was measured at 24 h using CCK-8 and flow cytometry (FITC Annexin V apoptosis Detection Kit, 556,547, BD). Besides, the cellular ROS were detected by the reactive oxygen species assay kit (S0033S, Beyotime).

### In vitro BMDCs stimulation experiment

According to an established method, Bone-marrow-derived DCs (BMDCs) were isolated from 6–8-week C57BL/6 mice. The PanC02 cells after different treatments (PBS, PBS + US, PPIX NDs, PPIX NDs + US, PMPS NDs, PMPS NDs + US) were incubated with BMDCs respectively. After 2-day stimulation, the BMDCs were DCs stained with anti-mouse CD11c BV421 (Biolegend, 117329), anti-mouse CD80 PE/cyanine7 (Biolegend, 104733), and anti-mouse CD86 BV785 (Biolegend, 105043) were analyzed by FCM (Beckman CytoFlex).

### In vitro HMGB-1 and CRT exploration

The PanC02 cells were cultured on a round coverslip or in 12-well plates. After different treatments (PBS, PBS + US, PPIX NDs, PPIX NDs + US, PMPS NDs, PMPS NDs + US), the cell was fixed using 4% paraformaldehyde at Day 2. The expression of HMGB-1 and CRT was evaluated by Western blot and immunofluorescence using anti-HMGB-1 (Abcam) and anti-CRT (Abcam). Moreover, the expression of CRT was also evaluated by FCM.

### The enhanced accumulation by cRGD and ADV in Vivo

To evaluate enhanced accumulation by cRGD and ADV effect, the 6–8-week C57BL/6 mice were subcutaneously inoculated with ${2\times 10}^{6}$ PanC02 cells. After the subcutaneous tumor reached 50–100 mm^3^, the mice were divided into different groups (NDs, cRGD NDs, NDs + US, cRGD NDs + US), and the nanoparticles were injected by tail vein. Then, the ADV groups (NDs + US and cRGD NDs + US) were irradiated using US (Frequency: 3 MHz; Acoustic intensity: 1.0 W/cm^2^; Duty cycle: 20%, 5 min) and took picture at 0.5, 2, 6, 24 h time point. At the last time point, the tumor and main organ were taken photo ex vivo.

### In Vivo DC maturing and for immune system activation study

Female C57BL/6 mice (6–8 weeks) were purchased from Shanghai SLAC Co. Ltd. All in vivo experiments were performed according to protocols approved by the Laboratory Animal Center of The Second affiliated Hospital of Zhejiang University School of Medicine. Mice were randomly divided into six groups (n = 5), including: (1) PBS, (2) PBS + US (3) PPIX NDs, (4) PPIX ND + US, (5) PMPS NDs and (6) PMPS NDs + US. PanC02 cells (${2\times 10}^{6}$) were orthotopically injected into the pancreas of each mouse. Seven days later, the tumors were allowed to reach 50 ~ 100 mm^3^ before the experiments. The mice in other groups were administered with PBS, PPIX NDs (PPIX 10 mg/kg), or PMPS NDs (equal dose of PPIX:10 mg/kg). In US irradiation group, US (Frequency: 3 MHz; Acoustic intensity: 1.0 W/cm^2^; Duty cycle: 20%; Times: 5 min) was conducted at five minutes and 6 h post-injection. On the third day, the tumor-draining lymph nodes were excised for analysis by FCM after co-staining with anti-mouse CD11c BV421 (Biolegend, 117329), anti-mouse CD80 PE/cyanine7 (Biolegend, 104733), and anti-mouse CD86 BV785 (Biolegend, 105043). Meanwhile, the blood samples were collected at 72 h after treatments, and the proinflammatory cytokines, including IL-6 and TNF-α, were tested by ELISA.

### Synergistic efficacy of ER-targeted SDT and aPD-1 for suppression of orthotopic and distant tumors

Firstly, the PanC02-luci cells (${2\times 10}^{6}$) were orthotopically injected into the pancreas of each mouse. Six days later, the PanC02-luci cells (${2\times 10}^{6}$) were subcutaneously injected into the right chest of each mouse. The tumor-bearing mice were divided into four groups randomly (N = 5), including:

(1) PBS, (2) aPD-L1, (3) PMPS NDs + US and (4) PMPS NDs + US + aPD-L1. The PBS and PMPS NDs were i.v. injected into animals at the same doses as that mentioned above on Days 0, 3, and 6. US irradiations that shared the identical parameters with those in the above experiments were performed after 0 and 6 h post-injection. aPD-L1 antibodies at the dose of 75 μg/mouse were administered on Days 0, 3, and 6. The fluorescence intensity of orthotopic tumor were measured using an in vivo imaging system, after injecting 100 µL (150 µg/mL) luciferin, and the subcutaneous tumor volume was calculated according to the following formula: (width^2^ × length)/2. At the end of the experiment, mice were sacrificed and tumors were excised, weighed and photographed.

### Mechanism investigation of the in vivo combined therapeutics

To systematically investigate the antitumor immune responses against orthotopic and distant tumors in vivo, the tumors were harvested and treated with the prepared tissue dissociation regent (collagenase 260 U/mL, Hyaluronidase 100 µg/mL, and DNase I 4 U/mL) to produce a single-cell suspension according to the specified procedures. The harvested cells were further stained with several fluorochrome-conjugated antibodies: Zombie Red™ Fixable Viability Kit (Biolegend, 423110), anti-mouse CD45 BV605 (Biolegend, 103139), anti-mouse CD3 PerCP/Cyanine5.5 (Biolegend, 100218), anti-mouse CD4 FITC (Biolegend, 100406), anti-mouse CD8 BV510 (Biolegend, 100752), anti-mouse CD49b APC (Biolegend, 108909), anti-mouse B220 BV421 (Biolegend, 103239) and then analyzed by FCM. All antibodies were diluted ~ 100 times.

### Immunohistochemistry and immunofluorescence assay

The orthotopic tumors were harvested in each group, and the H&E, TUNNEL, Ki-67 were stained to evaluate the antitumor efficacy. The expression of CRT was stained to evaluate the ICD.

### Statistical analysis

Data were expressed as means ± standard deviation (SD) and were compared by means of an unpaired Student’s t test, One-way ANOVA, Mann–Whitney U test, or Kruskal–Wallis test. All the statistical analyses were conducted using GraphPad software.

## Supplementary Information

**Additional file 1: ** Supporting information including additional methods and figures.

## Data Availability

The datasets used and/or analyzed during the current study are available from the corresponding author on reasonable request.

## Abbreviations

SDT: Sonodynamic therapy
ICD: Immunogenic cell death
ROS: Reactive oxygen species
CTLA-4: Cytotoxic T-lymphocyte-associated protein 4
PD-1: Programmed cell death protein-1
PD-L1: Programmed cell death ligand-1 (PD-L1)
IL: Interleukin
TEM: Transmission electron microscope
DAMPs: Damage-associated molecular patterns
ER: Endoplasmic reticulum
ADV: Acoustic droplets evaporation
PMPS NDs: ER-targeted sonodynamic droplets
PPIX: Protoporphyrin IX
MPSU: 4-Methylphenylsulfonylurea
TEA: Triethylamine (TEA)
BOP Reagent: (Benzotriazol-1-yloxy)tris(dimethylamino)phosphonium hexafluorophosphate
DLS: Dynamic light scattering
NDs: Nanodroplets
NMR: Nuclear magnetic resonance
UV–vis: Ultraviolet and visible
C5F10: Perfluoropentane
BMDCs: Bone marrow dendritic cells
FCM: Flow cytometry
CRT: Calreticulin
HMGB-1: High mobility group box-1
US: Ultrasound
aPD-L1: Anti-PD-L1;DOPE: 1,2-dioleoyl-sn-glycero-3-phosphoethanolamine
DSPE-PEG2000: 1,2-Distearoyl-sn-glycero-3-phosphoethanolamine-N-[methoxy(polyethylene glycol)-2000]
CHEMS: Cholesteryl hemisuccinate
ELISA: Enzyme-linked immunosorbent assay
H&E: Hematoxylin and eosin stain
TUNNEL: TdT-mediated dUTP nick end labeling
ANOVA: Analysis of variance
