# Supplementary material for: Cavitation assisted endoplasmic reticulum targeted sonodynamic droplets to enhanced anti-PD-L1 immunotherapy in pancreatic cancer
Source: J Nanobiotechnology. 2022 Jun 16;20:283. doi: 10.1186/s12951-022-01459-w (PMC9202099; doi:10.1186/s12951-022-01459-w)
Supplement: Supplementary file 1 — Additional file 1: Supporting information including additional methods and figures. [file 12951_2022_1459_MOESM1_ESM.docx]

Supporting Information

**Cavitation Assisted Endoplasmic Reticulum Targeted Sonodynamic Droplets to enhanced Anti-PD-L1 Immunotherapy in Pancreatic Cancer.**

*Jifan Chen^1,3#^, Liting Feng^4,#^, Peile Jin^1,3#^, Jiaxin Shen^1,3^, Jiayue Lu^5^, Yue Song^1,3^, Guowei Wang^1,3*^, Qin Chen^4^, Deyi Huang^6^, Ying Zhang^1,3^, Chao Zhang^1,3^, Youfeng Xu^2*^, Pintong Huang^1,3,7*^*

1. Department of Ultrasound in Medicine, The Second Affiliated Hospital of Zhejiang University School of Medicine, Zhejiang University, Hangzhou, 310000, China;
2. Department of Ultrasound, Ningbo First Hospital, Ningbo, 315000, China.
3. Research Center of Ultrasound in Medicine and Biomedical Engineering, The Second Affiliated Hospital of Zhejiang University School of Medicine, Zhejiang University, Hangzhou, 310009, China.
4. Department of Ultrasound, Sichuan Provincial People's Hospital, University of Electronic Science and Technology of China, Chengdu, 610000, China.
5. Department of Clinical Laboratory, Second Affiliated Hospital, Zhejiang University School of Medicine, Hangzhou, 310009, China.
6. Department of Ultrasound, Yuhuan People’s Hospital, Taizhou, 317600, China.
7. Research Center for Life Science and Human Health, Binjiang Institute of Zhejiang University, Hangzhou, 310053, China.

^#^These Authors Contribute Equally.

^*^Corresponding Author. (huangpintong@zju.edu.cn, xuyoufeng2017@163.com, wangguowei@zju.edu.cn)

Corresponding Author:

*Pintong Huang*

Department of Ultrasound in Medicine, The Second Affiliated Hospital of Zhejiang University School of Medicine, Zhejiang University, Hangzhou, 310000, China;

E-Mail: huangpintong@zju.edu.cn

*Youfeng Xu*

Department of Ultrasound, Ningbo First Hospital, Ningbo 315010, China

E-Mail: xuyoufeng2017@163.com

*Guowei Wang*

Department of Ultrasound in Medicine, The Second Affiliated Hospital of Zhejiang University School of Medicine, Zhejiang University, Hangzhou, 310000, China;

E-Mail: wangguowei@zju.edu.cn


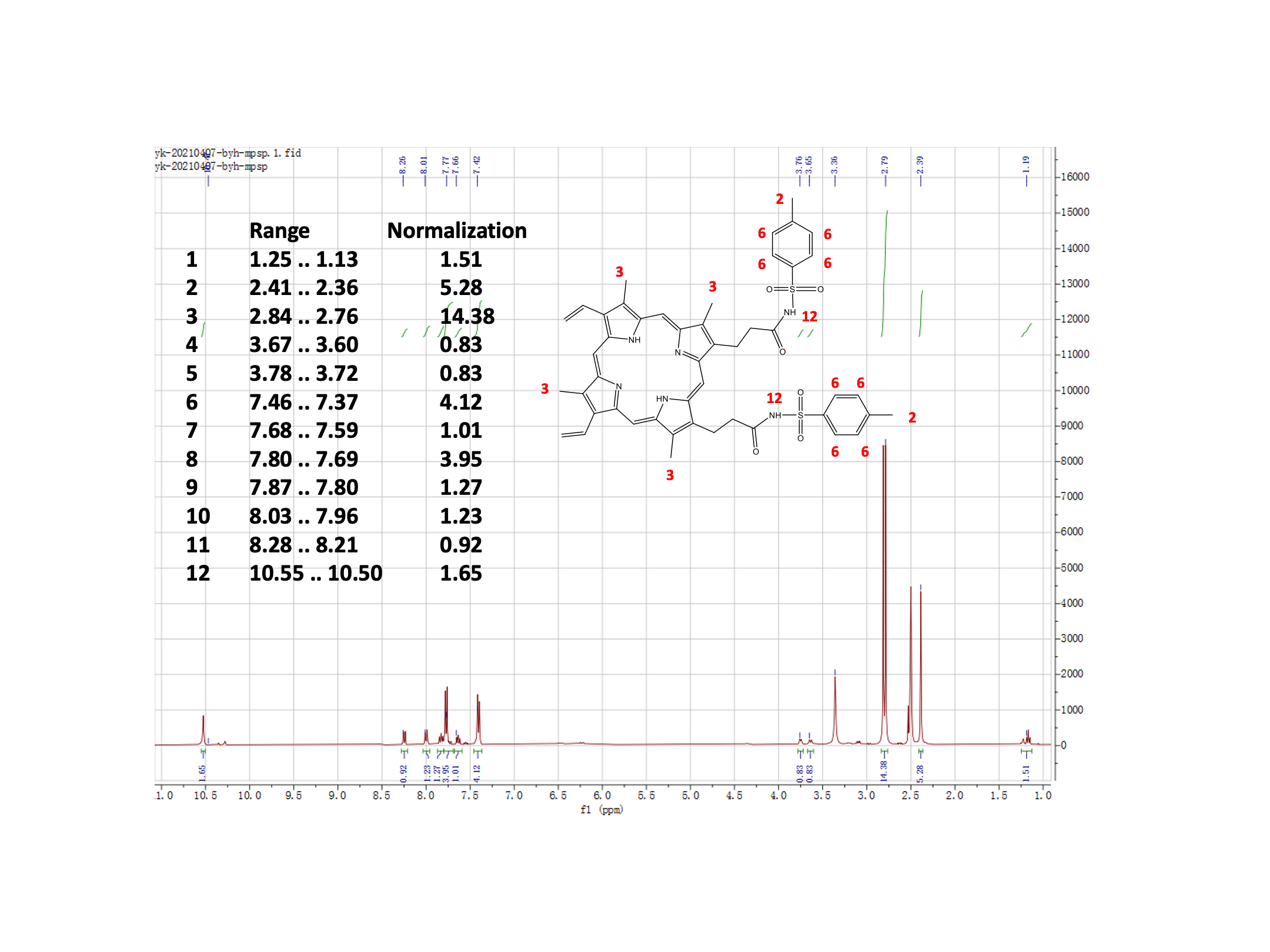


**FigureS1: The nuclear magnetic resonance spectrum of PMPS.**


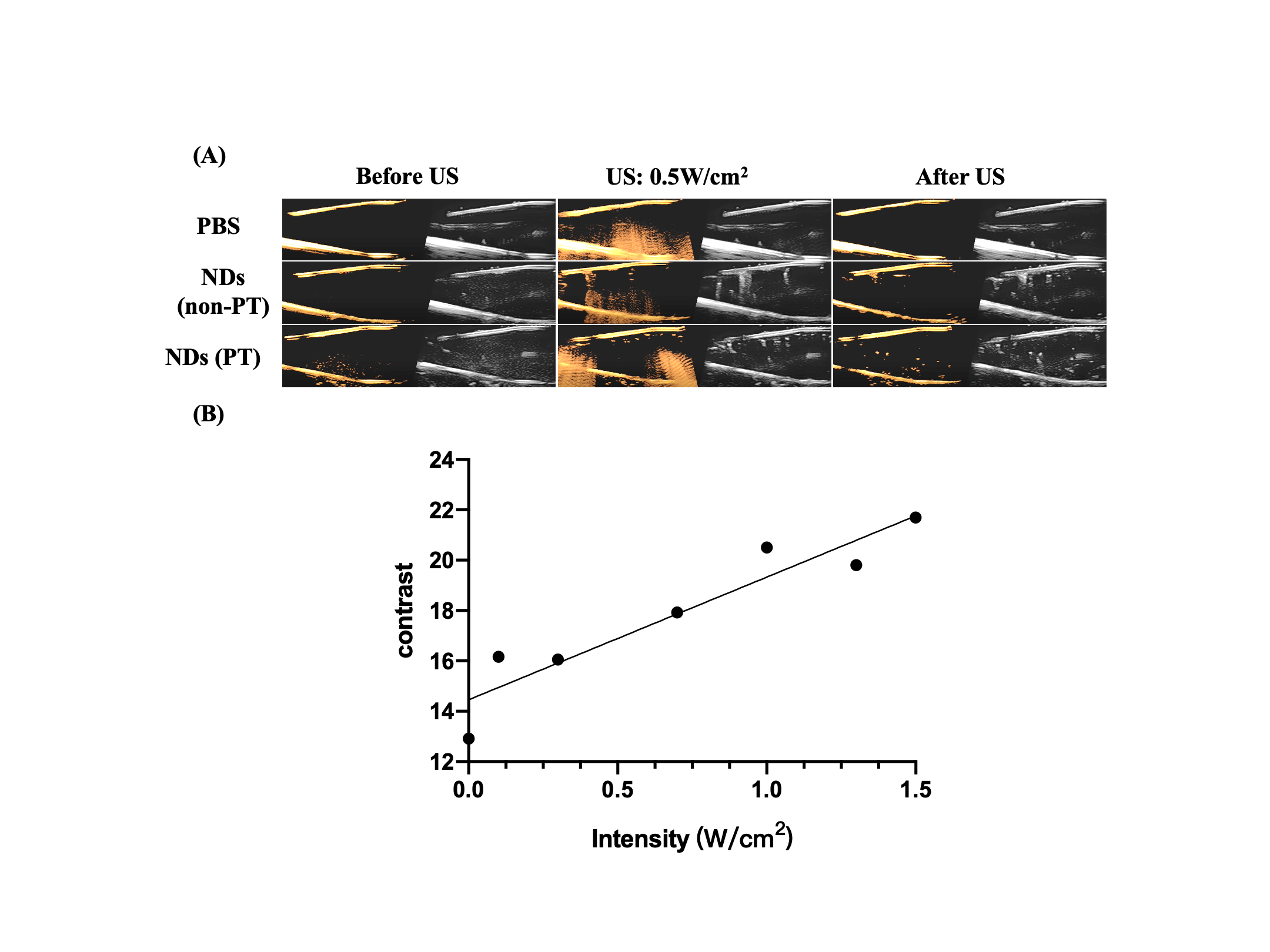


**FigureS2: The droplet evaporation effect:** the acoustic droplet evaporation (A & B)


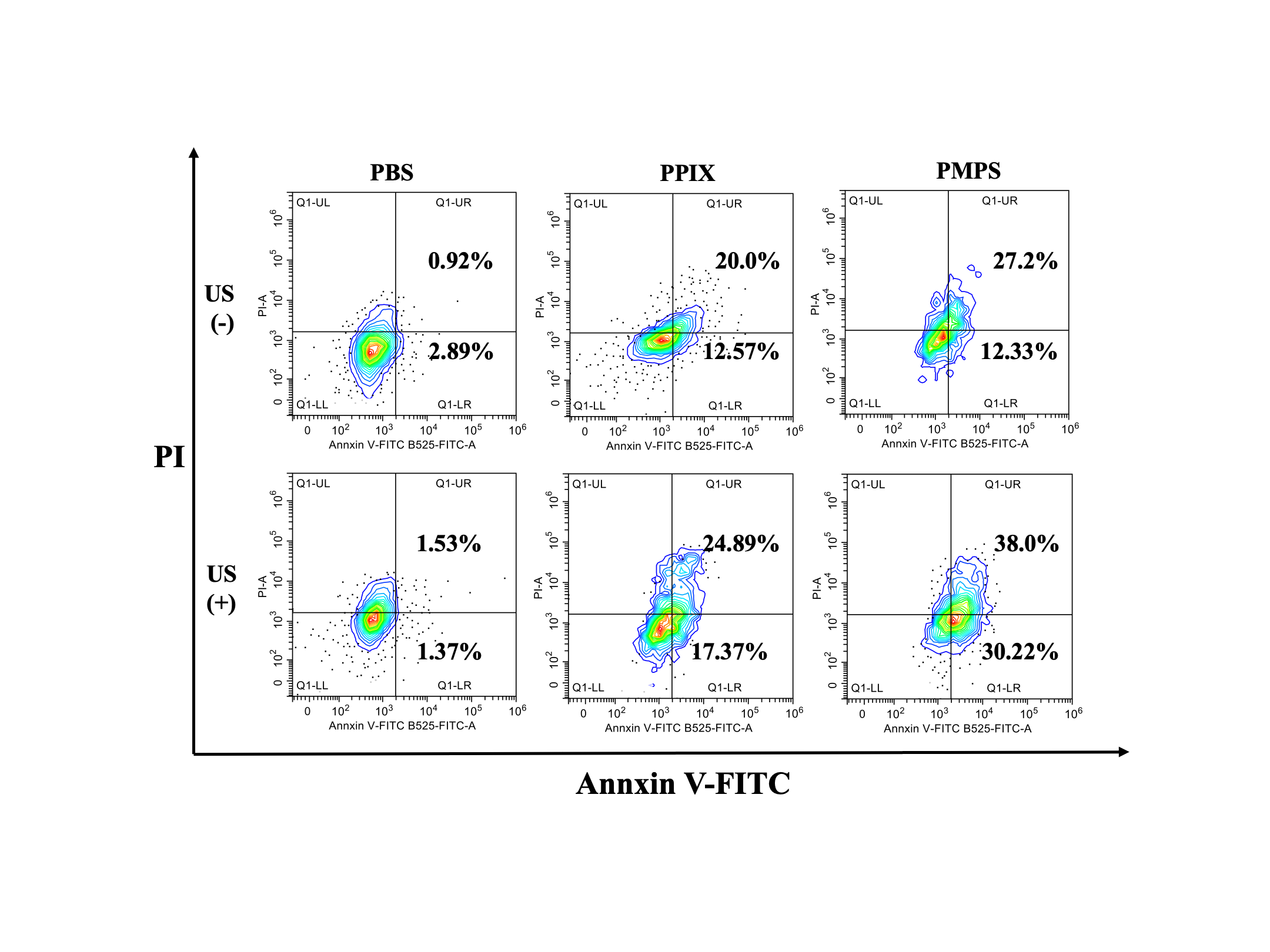


**FigureS3: the Apoptosis effect using FCM.**


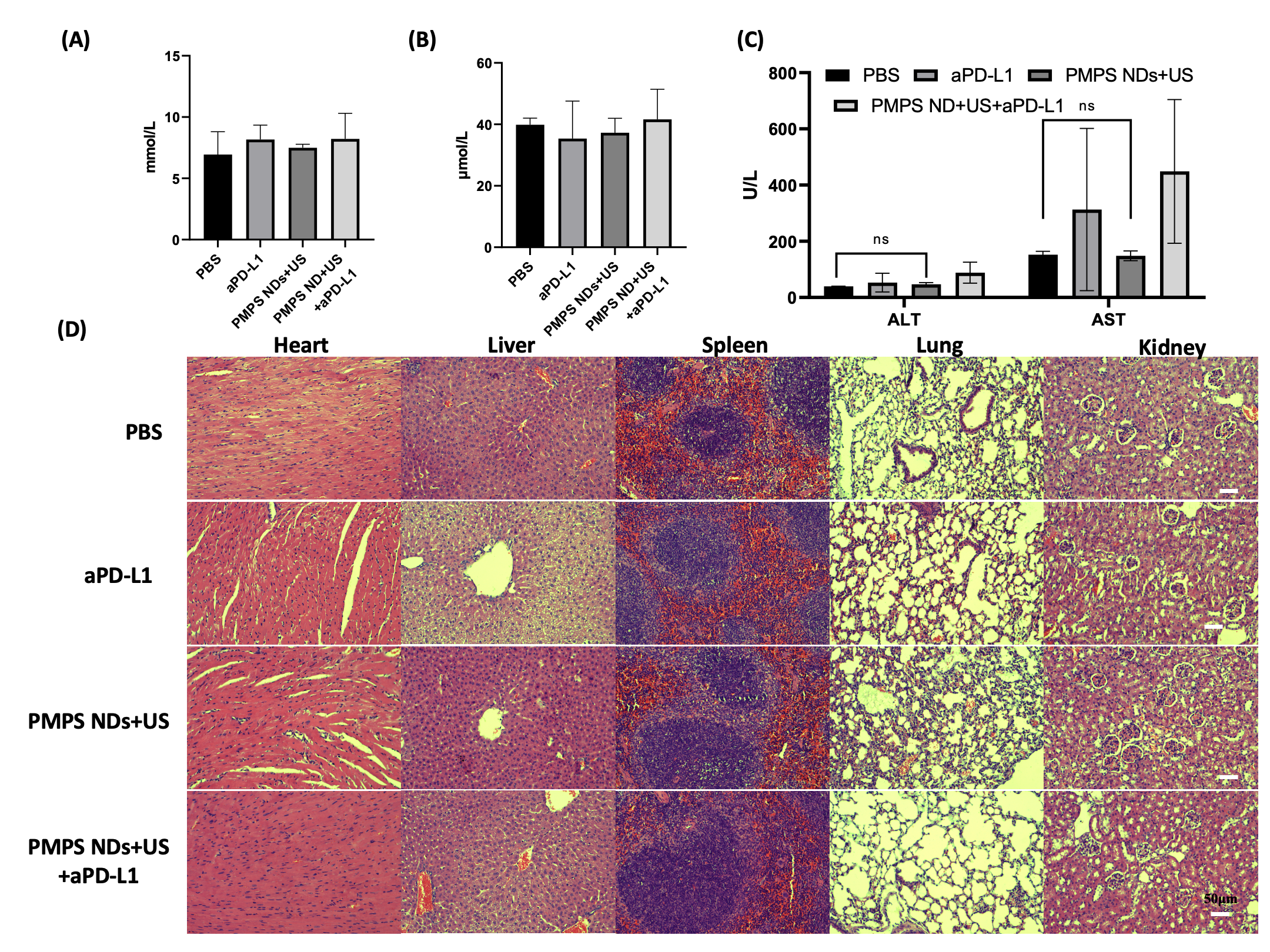


**FigureS4: the biosafety evaluation of PMPS NDs.** The biochemical index in serum of BUN (A), CR (B) and ALT and AST(C). The H&E of main organ (Heat, Liver, Spleen, Lung and Kidney) (D).


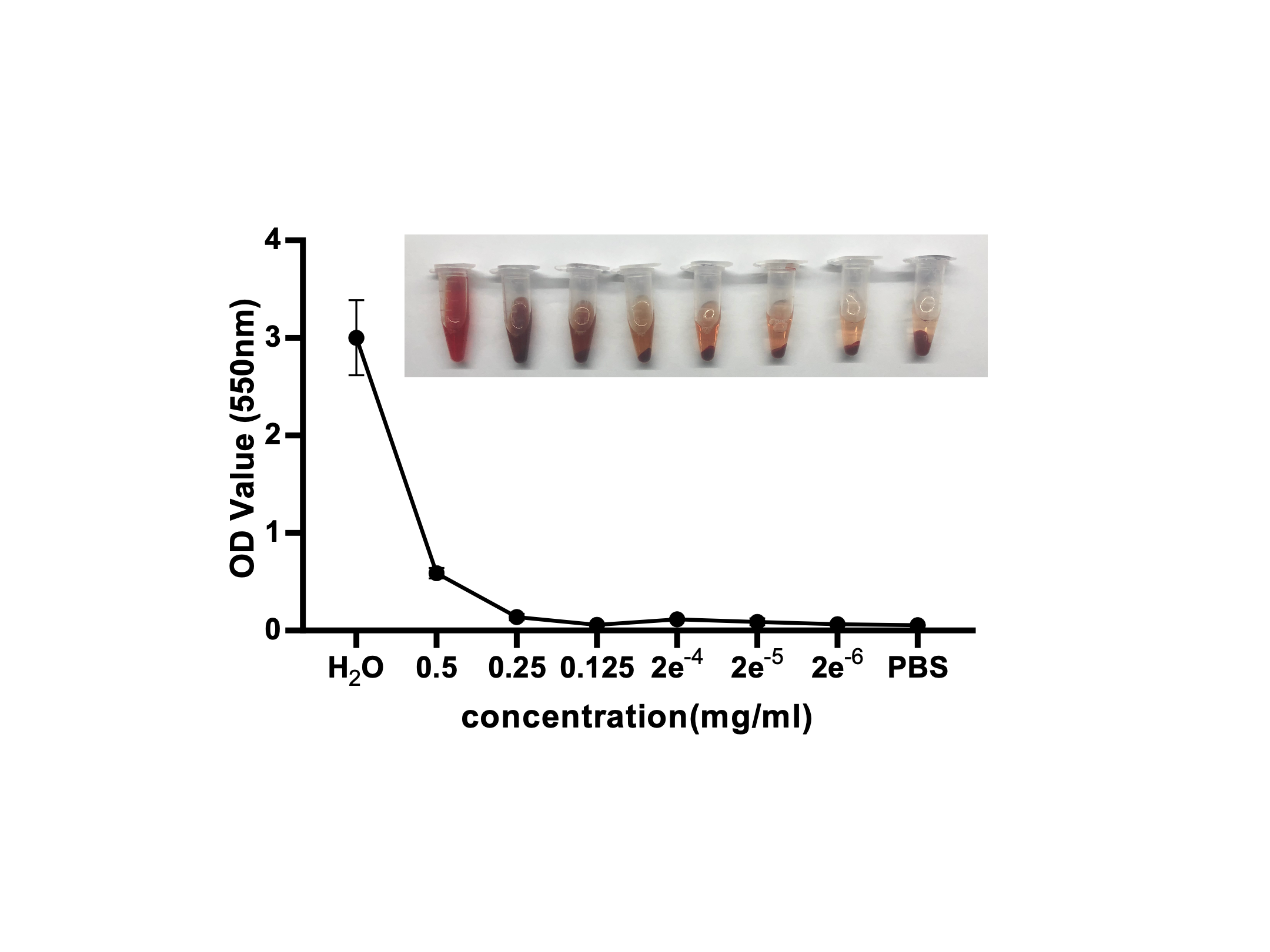


**FigureS5: The hemolysis of PMPS NDs.**


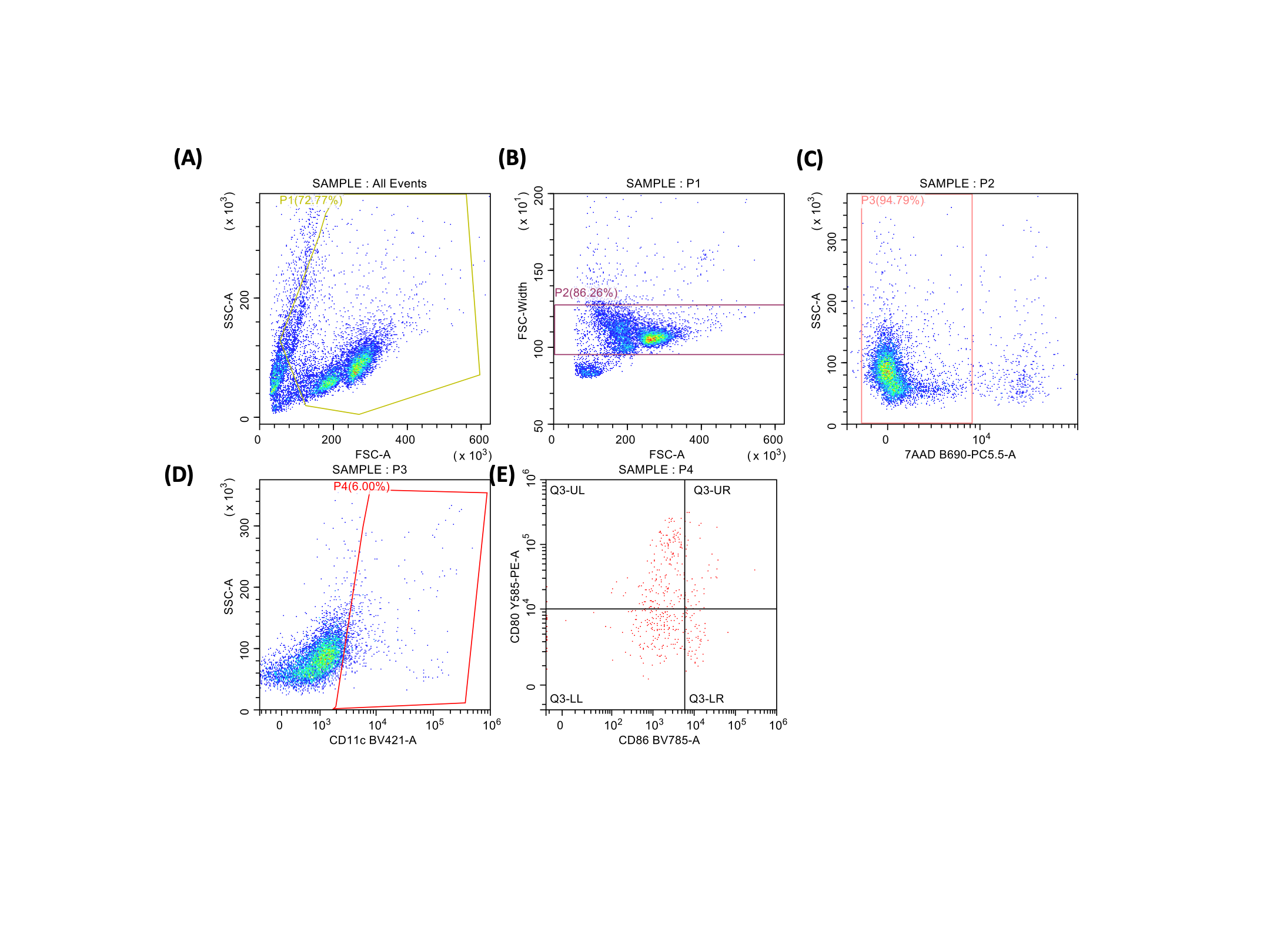


**Figure S6: The protocol of flow cytometry of DC**: Assessed by flow cytometry after choosing by SSC-A and FSC-A (A), avoiding cytoadherence(B), staining with live dead(C), CD11c (D), CD80 and CD86 (E).


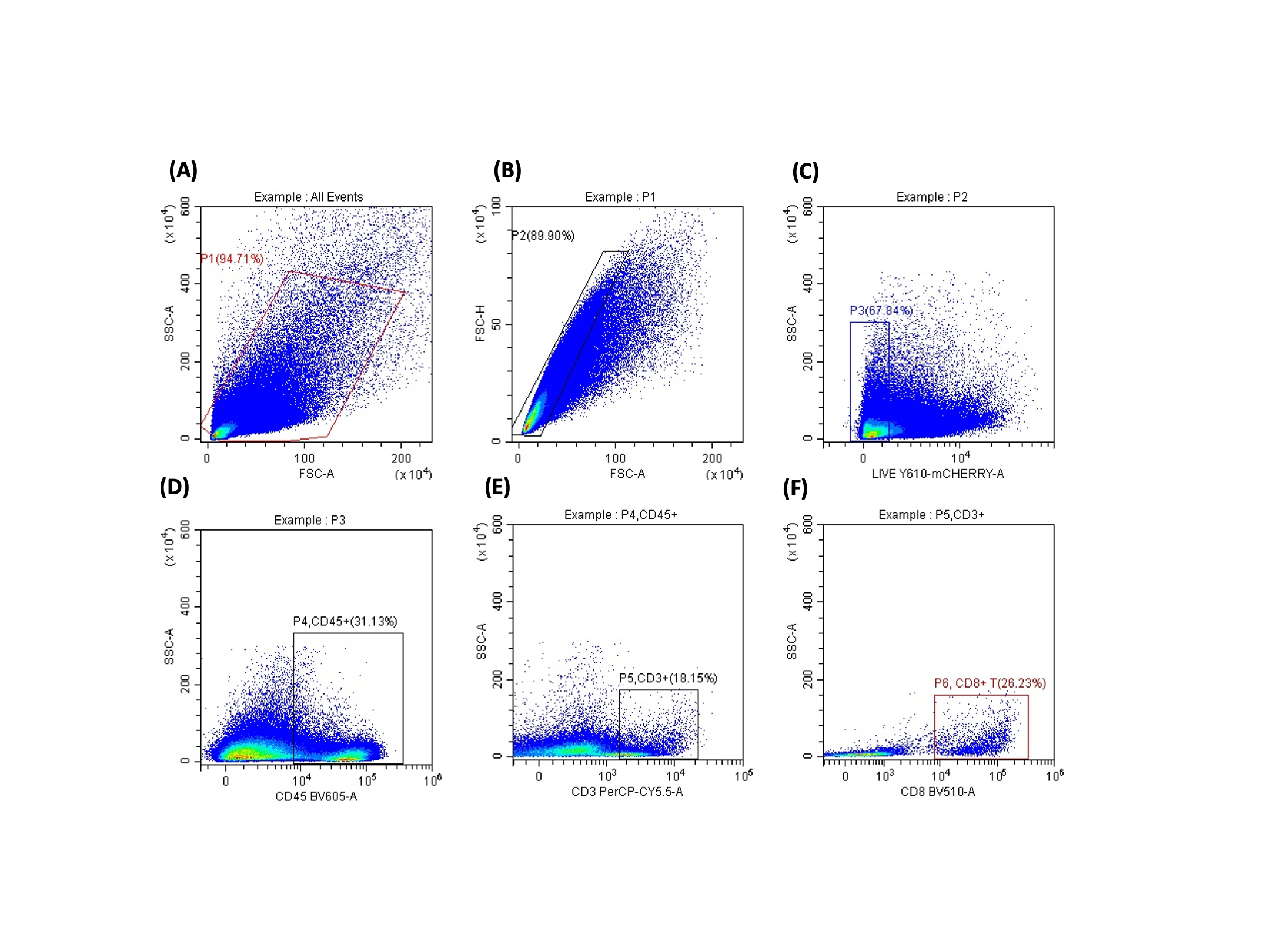


**Figure S7:** The protocol of flow cytometry of CD8+ T cell: Assessed by flow cytometry after choosing by SSC-A and FSC-A (A), avoiding cytoadherence (B), staining with live dead(C), CD45 (D), CD3 (E) and CD8 (F).


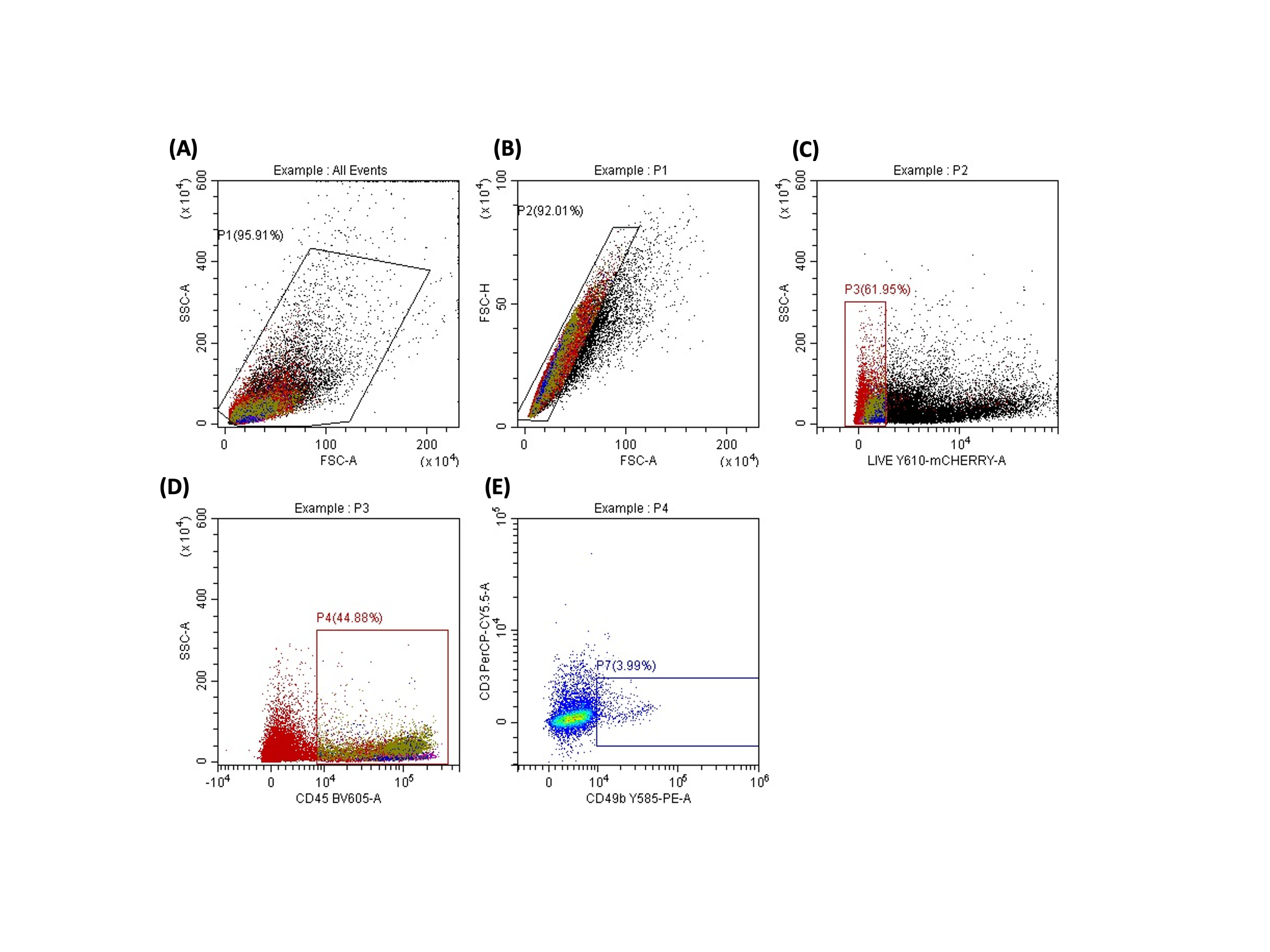


**Figure S8: The protocol of flow cytometry of NK cell:** Assessed by flow cytometry after choosing by SSC-A and FSC-A (A), avoiding cytoadherence (B), staining with live dead(C), CD45 (D), CD3 and CD49b (E).
